# Transforming Growth Factor-****β****1 Gene Polymorphism (T29C) in Egyptian Patients with Hepatitis B Virus Infection: A Preliminary Study

**DOI:** 10.1155/2013/293274

**Published:** 2013-12-18

**Authors:** Roba M. Talaat, Mahmoud F. Dondeti, Soha Z. El-Shenawy, Omaima A. Khamiss

**Affiliations:** ^1^Molecular Biology Department, Genetic Engineering and Biotechnology Research Institute (GEBRI), University of Sadat City, Sadat City 22857, Egypt; ^2^Biochemistry Department, National Liver Institute (NLI), Menoufiya University, Shebeen El-Kom, Menoufiya 32511, Egypt; ^3^Animal Biotechnology Department, Genetic Engineering and Biotechnology Research Institute (GEBRI), University of Sadat City, Sadat City 22857, Egypt

## Abstract

The interindividual variations in the capacity of transforming growth factor-**β**1 (TGF-**β**1) production have been ascribed to genetic polymorphisms in TGF-**β**1 gene. As pathogenesis of HBV has a genetic background, this preliminary study was designed to assess the impact of TGF-**β**1 (T29C) on the susceptibility of Egyptians to HBV infection. Genotyping was performed using single stranded polymorphism-polymerase chain reaction (SSP-PCR) in 65 Egyptian hepatitis B patients and 50 healthy controls. TGF-**β**1 plasma levels were measured using Enzyme-linked immunosorbent assay (ELISA). The frequency of CC genotype was significantly higher (*P* < 0.05) in HBV patients compared to controls. On the contrary, TC genotype did not show significant difference in both groups. TT genotype was significantly higher (*P* < 0.01) in controls than HBV patients. Our current preliminary data revealed that the frequency of the genotypes in the controls were within Hardy-Weinberg equilibrium (HWE) while the patients group was out of HWE (*P* < 0.01). TGF-**β**1 was significantly (*r* = −0.684; *P* < 0.001) deceased in the sera of patients as compared to normal subjects. Depending on our preliminary work, CC genotype may act as a host genetic factor in the susceptibility to HBV infection in Egyptians. Taken together, the current data pointed to the importance of polymorphism of TGF-**β**1 gene (T29C) in HBV infection.

## 1. Introduction

Hepatitis B virus infection (HBV) is a worldwide problem and it is still the main factor of developing chronic HBV, cirrhosis, and hepatocellular carcinoma (HCC), especially in developing countries [1]. There are about 400 million carriers of HBV infection worldwide and over 1 million deaths occur each year as a consequence of fulminant hepatic failure, cirrhosis, and hepatocellular carcinoma [2]. Moreover, 5–10% of infected individuals cannot clear the infection, which leads to a chronic carrier state with or without liver disease chronic [3]. The interaction of the host immune response with HBV, the impact of this interaction on the clinical outcome, and the factors of viral persistence are not yet fully understood. Host genetic factors have been reported to be critical factors which affect the natural history of liver diseases [4].

Transforming growth factor-*β*1 (TGF-*β*1) is a multifunctional cytokine that regulates cell growth, proliferation, and differentiation [5]. It is produced by several cell types, including monocytes, macrophages, endothelial cells, and vascular smooth muscle cell [6, 7] and it is also produced from a variety of liver cell populations including HSCs, hepatocytes, and LSECs in addition to platelets and infiltrating mononuclear cells [8, 9]. TGF-*β*1 is key molecule in many physiological processes in the liver since it induces apoptosis and reduces hepatocytes proliferation besides its essential role in hepatic fibrogenesis. Host genetic factors play a critical role in developing fibrosis whereas many genes are reported to be associated with liver fibrosis and cirrhosis including TGF-*β*1 [10]. In addition, TGF-*β*1 has potential impact on the immune response since it has immunosuppressive effects like its inhibitory effect on T-cells proliferation via IL-2 down-regulation [11]. TGF-*β*1 gene is located on chromosome 19q13.1–13.3 with 7 exons and 6 introns [12, 13]. Several polymorphisms in both coding and non-coding regions of the TGF-*β*1 gene have been reported and found to affect TGF-*β*1 protein expression [14]. There is a functional single nucleotide polymorphism (SNP) at the 29th nucleotide (T29C), 868 nt relative to the transcription start site, (rs1982073 merged into rs1800470) in exon 1 with transition from T to C resulting in amino change in the region encoding the signal sequence from Leucine to Proline at the 10th amino acid [14–16]. This transition disrupts the structure [17, 18] and results in increased levels of TGF-*β*1 protein and mRNA in individuals with C allele with a 2.8-fold increase in TGF-*β*1 secretion compared with T allele in vitro [16, 19–21]. Additionally, the substitutions of amino acid residue might affect the function of the signal peptide, possibly by influencing intracellular trafficking or export efficiency of the TGF-*β*1 protein [20]. It was also reported that C allele of 29T/C is associated with increased TGF-*β*1 serum levels, thereby the T29C polymorphism maybe influence the development and severity of TGF-*β*1-related diseases and it has been associated with susceptibility to several diseases [7, 20, 22, 23]. Thus, this preliminary study was tailored to investigate the role of TGF-*β*1 gene (T29C) in HBV infection in Egyptians. No such study has been conducted to investigate the association between SNP in TGF-*β*1 gene (T29C) and HBV infection in Egypt.

## 2. Materials and Methods

### 2.1. Patients and Controls

Sixty five patients with chronic HBV infection were recruited from the National Liver Institute, Menoufiya University, Egypt, were enrolled in this study. The males over numbered the females (53 men and 12 women) with mean age of 44.93 ± 11.57 years (range: 68–22). The demographic and biochemical characteristics are presented in Table 1. Fifty healthy controls with no history of previous liver disease, normal liver function tests, and negative HBV and HCV serology were enrolled in the study. Patients with HCV or other viral infections or any liver diseases were excluded from the study. All investigations were performed in accordance with the Menoufiya University, Health and Human Ethical Clearance Committee guidelines for Clinical Researches. Local Ethics Committee approved the study protocol and informed consents were got from all subjects.

### 2.2. Viral Assessment

Hepatitis B surface antigen (HBsAg) was tested using a commercial kit (Sorin Biomedica, Milan, Italy) while HBV-DNA in HBV-positive patients was tested by polymerase chain reaction (PCR), (Roche Diagnostics Corp., Indianapolis, IN). HCV antibodies were tested by using enzyme-linked immunosorbent assay (ELISA) (Murex Biotech Ltd., Dartford, UK) All patients were positive for HBsAg, HBV-DNA, and negative for HCV antibodies. Alanine aminotransferase (ALT), aspartate aminotransferase (AST) (bioMérieux S.A, Marcy l'Etoile, France), direct and indirect bilirubin (Roche Diagnostics Corp., Indianapolis, IN), and albumin (Human Gesellschaft Fur Biochemica Und Diagnostica Mbh, Wiesbaden, Germany) were all measured according to their respective kits' manufacturers' instructions.

### 2.3. DNA Isolation

Blood samples were collected by withdrawal of 5 mL venous blood from each individual involved in this study into sterile vacutainer tubes containing EDTA.K_3_, and then the tubes were centrifuged at 1500 rpm for 10 minutes. Plasma was separated, aliquoted, and stored at −80°C for cytokine secretion analysis. Genomic DNA was extracted from whole blood-EDTA samples by Wizard Genomic DNA Purification Kit (Promega Corporation, Madison, USA) according to manufacturer's instructions.

### 2.4. Genotyping

**Figure 1:**
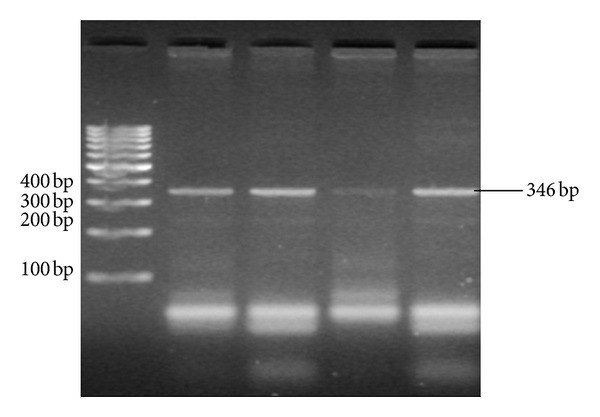
TGF-*β*1 (T29C) PCR products of two samples. Sample 1 in lane (2 and 3) TC genotype; sample 2 in lane (4 and 5) CC genotype and lane (1) 100 bp ladder.

TGF-*β*1 T29C was genotyped by single stranded polymorphism-polymerase chain reaction (SSP-PCR) [24] using the following primers: T allele specific primer 5-CTCCGGGCTGCGGCTGCTGCT-3, C allele specific primer 5-CTC CGG GCT GCG GCT GCT GCC-3, and reverse common primer 5-GTT GTG GGT TTC CAC CAT TAG-3 [15]. The PCR reaction was performed in two tubes in which each tube contains forward primer specific to one allele in addition to generic primer. The final total volume for each PCR reaction was 25 *μ*L. PCR reaction ingredients were DreamTaq Green Master Mix 2x (Fermentas, Thermo Fisher Scientific Inc.), 10 P moles of each primer (Metabion, Martinsried, Deutschland) and 0.1 *μ*g DNA. The PCR cycling was the following; one cycle of 94°C for 5 minutes followed by 35 cycles of 96°C for 30 seconds, 59°C for 30 seconds, 72°C for 55 seconds, and a final extension step of 5 minutes. PCR reaction was performed in Biometra thermal cycler (Biometra GmbH, Germany). The PCR products were visualized on 2% agarose gel and estimated in comparison to 100 bp DNA ladder (Fermentas, Thermo Fisher Scientific Inc.). The size of PCR product for TGF-*β*1 T29C primers was 346 bp for T or C allele (Figure 1).

### 2.5. Measurement of Plasma TGF-*β*1

TGF-*β*1 plasma levels were measured in HBV patients and normal controls by sandwich enzyme linked immunosorbent assay (ELISA) (R&D System, Inc., Minneapolis, USA) according to manufacturer's instructions. The ELISA reader-controlling software (Softmax, Molecular Devices Corp., USA) readily processed the digital data of raw absorbance value into a standard curve from which cytokine concentrations of unknown samples can be derived directly and expressed as pg/mL.

### 2.6. Statistical Analysis

The statistical analyses were performed by SPSS statistical package version 19 (SPSS, IBM Corporation, USA). Comparisons were made using independent *t*-test and results were presented as mean ± SD. Chi-squared tests were performed to examine the differences in the allele frequency and genotype distribution between different groups. Odds ratios (with 95% CI) were calculated to measure the relative risks in both control and HBV patients. All *P* values were two-tailed, and *P* values <0.05 were considered to be statistically significant.

## 3. Results 

### 3.1. Association between TGF-*β*1 Gene (T29C) Polymorphism and Hepatitis B Infection

TGF-*β*1 T29C genotypes and allele frequencies in controls and patients are shown in (Table 2). The frequency of TGF-*β*1 (T29C) genotypes in the controls were within Hardy-Weinberg equilibrium (HWE) while they were out of HWE (*P* < 0.01) in the patients group. Genotyping of TGF-*β*1 T29C showed a significant decrease (*P* < 0.01) in the distribution of TT genotype in controls in comparison to HBV patients (44.4% versus 16.9%, for control and HBV, resp.). While CC genotype was not detected in the control group while it appeared in the patient group with a percentage of 15.4%. On the contrary, the frequency of TC genotype was insignificantly different in normal controls compared with HBV patients. C allele was significantly (*P* < 0.01) more frequent in HBV patients more than controls groups while distribution of T allele did not show significant difference between both groups (92.6%, 84.6% for T allele versus 55.6%, 83.1% for C allele in control and HBV, resp.).

### 3.2. Plasma Levels of TGF-*β*1 in HBV and Normal Controls and Its Differential Expression according to TGF-*β*1 T29C

Mean plasma levels were significantly lower (*P* < 0.001) in HBV patients than controls (63.48 ± 7.59 pg/mL versus 12151.76 ± 2124.90 pg/mL). Hepatitis B was significantly correlated with a reduction in TGF-*β*1 plasma levels (*r* = −0.684; *P* < 0.001). The comparisons between concentration of plasma TGF-*β*1 levels with different genotypes in both controls and HBV patients are shown in (Table 3). The reduction in TGF-*β*1 secretion levels, observed in HBV patients compared to normal controls, were relevant to TGF-*β*1 T29C genotypes. Thus, CC genotype was responsible for the significant decrease of TGF-*β*1 level between both groups while TT genotype was relevant to high TGF-*β*1 serum level.

## 4. Discussion

Genetic susceptibility to chronic HBV infection and other infectious diseases may reside in the variability in host recognition, cytokine, or antigen presenting and processing genes [25]. Since genetic interactions are complex, it is unlikely that a single allelic variant is responsible for HBV resistance or susceptibility [26]. The ongoing study of the distributions and functions of the implicated allele polymorphisms will not only provide insight into the pathogenesis of HBV infection, but may also provide a novel rationale for new methods of diagnosis and therapeutic strategies [27]. The aim of studying such polymorphisms and their association with diseases is to enhance the understanding of the etiology and pathology of human disease, and to identify potential markers of susceptibility, severity, and clinical outcome, and to identify potential markers for responders versus nonresponders in therapeutic trials, and to identify targets for therapeutic intervention, in addition to identify novel strategies to prevent disease or to improve existing preventions such as vaccines [28]. The majority of the human genetic studies associated with HBV infection have focused on HLA associations [29, 30]. There are many studies of host genetic factors especially cytokine genes that influence the immune response mounted against HBV infection [31]. In addition, Cytokine gene polymorphisms have been reported to be associated with liver disease severity in patients with viral hepatitis [10, 25] besides their impact on the cytokines production capacity [32–34]; therefore, heterogeneity of the candidate gene in HBV-infected patients serves as a probable biomarker for influence the disease phenotypes.

Several polymorphisms located in genes that code pro- and anti-inflammatory molecules have been reported to be associated with HBV infection. Moreover, more light was shed on TGF-*β*1 since it is a central regulator in immuno-inflammatory mechanisms. Therefore, this preliminary was conducted to investigate the role of polymorphisms in TGF-*β*1 gene and HBV infection among Egyptians. In the present preliminary study TGF-*β*1 T29C was studied in 65 Egyptian hepatitis B patients and 50 healthy controls. The common T29C transition in TGF-*β*1, resulting in a Leu10Pro substitution in the signal peptide sequence, is a good candidate locus because it has been associated with higher levels of circulating TGF-*β*1 [16, 22] especially the presence of the C allele in exon 1 results in increasing the production of TGF-*β*1 [7, 16, 19, 35, 36]. Interestingly, Gewaltig et al., reported that the presence of C allele at TGF-*β*1 (T29C) either C/C or C/T genotype, was associated with higher stage of fibrosis in HCV-infected patients [10].

Our preliminary results showed that TT genotype, which was more frequent in controls than HBV patients, was associated with higher significant levels of serum TGF-*β*1. Additionally, the plasma levels were significantly higher in the controls more than the patients and this is contradictory to previous results which showed that T allele was associated with lower level of TGF-*β*1 [15, 20, 22, 37] and C allele was associated with higher TGF-*β*1 levels [38, 39]. On the other hand, our preliminary results were consistent with other studies of [40, 41] in which TGF-*β*1 serum levels were lower in the subjects with the CC homozygote than in those with the TT homozygote at T29C of TGF-*β*1 gene. According to our preliminary study and previous studies, there is momentous discrepancy in the revealed results but this can be attributed to the differential genetic background of investigated populations and the different ethnicity of studied populations. In addition, there are other studies which have failed to reveal any association between serum TGF-*β*1 levels and SNP at TGF-*β*1 (T29C) [41, 42]. In conclusion, our preliminary results may suggest the protective role of TT genotype against HBV infection and this will need to be confirmed further in a large study. On the other hand, CC genotype was hardly detected in controls and this may indicate the involvement of this genotype in the susceptibility of HBV infection and consequences.

There is shortcoming in the current study that it depended on one group of cases in addition to the number of subjects enrolled in the study and this made the elucidation of some results a hard and onerous task as we cannot confirm the relationship between the current findings and the progression of the infection, so the study had to include other categories of the disease as groups of cirrhotic and HBV patients with HCC. Studying genetic polymorphisms in TGF-*β*1 gene will clear the precise role of TGF-*β*1 in the pathogenesis of HBV infection and its role in the susceptibility to HBV infection as TGF-*β*1 has a deep impact on the immune response. Therefore, we are performing genotyping of TGF-*β*1 gene in large number of patients and studying many SNPs as TGF-*β*1-800G/A, TGF-*β*1-509C/T, TGF-*β*1+869T/C, and TGF-*β*1+915G/C to study the relation between TGF-*β*1 gene polymorphism and HBV infection and to confirm the current preliminary results.

## Figures and Tables

**Table 1:** Demographic and biochemical characteristics of HBV patients and healthy controls.

Parameter	Control group (*N* = 65)	HBV group (*N* = 50)	*P *	Correlation with disease
Demographic data				
Age (mean ± SD)	44.92 ± 11.76	32.11 ± 14.89	NS	*r* = 0.437 *P* < 0.001
Gender (Male ♂: Female ♀)	53/12 (81.5/18.5%)	14/36 (28%/72%)	*P* < 0.001	*r* = 0.523 *P* < 0.001

Laboratory investigations (mean ± SD)				
AST (IU/L)	41.59 ± 3.47	22.18 ± 1.05	*P* < 0.001	*r* = 0.473 *P* < 0.001
ALT (IU/L)	44.49 ± 5.61	16.74 ± 0.86	*P* < 0.01	*r* = 0.455 *P* < 0.001
Albumin (g/L)	3.37 ± 0.12	4.35 ± 0.07	*P* < 0.01	*r* = −0.625 *P* < 0.001
Total bilirubin (mg/dL)	1.03 ± 0.08	0.70 ± 0.04	*P* < 0.05	*r* = 0.381 *P* < 0.01
Direct bilirubin (mg/dL)	0.25 ± 0.06	0.12 ± 0.03	*P* < 0.05	*r* = 0.198 NS
Creatinine (mg/dL)	1.11 ± 0.06	0.89 ± 0.03	*P* < 0.01	*r* = 0.332 *P* < 0.01
Urea (mg/dL)	33.16 ± 2.26	29.85 ± 1.41	*P* < 0.01	*r* = 0.130 NS
HBV DNA (IU/L)	—	1003076.02 ± 914392.11	—	*r* = 0.168 *P* < 0.05

All data are presented as mean ± SD. Alanine aminotransferase (ALT); Aspartate aminotransferase (AST).

**Table 2:** Genotype and allelic frequencies of the TGF-*β*1 T29C in patients with hepatitis B and healthy controls.

SNP	HBV group (*N* = 65)	Control group (*N* = 50)	*P *	OR (95% CI)
Genotype frequency (*N*, %)				
T/T	11 (16.9%)	12 (44.4%)	*P* < 0.01	0.2546 (0.0938–0.6910)
T/C	44 (67.7%)	15 (55.6%)	NS	1.6762 (0.6680–4.2062)
C/C	10 (15.4%)	0 (0.0%)	*P* < 0.05	10.4054 (0.5878–184.1951)
TCCC	54 (83.1%)	15 (55.6%)	*P* < 0.05	3.2000 (1.1697–8.7541)

Allele frequency				
T	66 (84.6%)	39 (92.6%)	NS	0.3966 (0.1994–0.7889)
C	64 (83.1%)	15 (55.6%)	*P* < 0.01	2.5212 (1.2676–5.0147)

**Table 3:** Comparison between mean serum concentrations of TGF-*β*1 according to TGF-*β*1 T29C in hepatitis B patients and healthy controls.

Genotype (Control, HBV)	Control group (*N* = 65)	HBV group (*N* = 27)	*P *
T/T (11, 12)	11577.47 ± 3111.76	83.89 ± 21.15	P < 0.001
T/C (44, 15)	1289.61 ± 2919.49	63.72 ± 9.46	P < 0.001
C/C (10, 0)	—	39.94 ± 10.21	P < 0.001
